# Supplementary material for: Measuring the distribution of fitness effects in somatic evolution by combining clonal dynamics with dN/dS ratios
Source: eLife. 2020 Mar 30;9:e48714. doi: 10.7554/eLife.48714 (PMC7105384; doi:10.7554/eLife.48714)
Supplement: Transparent reporting form [file elife-48714-transrepform.docx]

***eLife’s* transparent reporting form**

We encourage authors to provide detailed information *within their submission* to facilitate the interpretation and replication of experiments. Authors can upload supporting documentation to indicate the use of appropriate reporting guidelines for health-related research (see [EQUATOR Network](http://www.equator-network.org/%20)), life science research (see the [BioSharing Information Resource](https://biosharing.org/)), or the [ARRIVE guidelines](http://www.plosbiology.org/article/info:doi/10.1371/journal.pbio.1000412) for reporting work involving animal research. Where applicable, authors should refer to any relevant reporting standards documents in this form.

If you have any questions, please consult our Journal Policies and/or contact us: [editorial@elifesciences.org](mailto:editorial@elifesciences.org).

**Sample-size estimation**

- You should state whether an appropriate sample size was computed when the study was being designed
- You should state the statistical method of sample size computation and any required assumptions
- If no explicit power analysis was used, you should describe how you decided what sample (replicate) size (number) to use

Please outline where this information can be found within the submission (e.g., sections or figure legends), or explain why this information doesn’t apply to your submission:

We used publicly available for our analysis.

We used normal sequencing data from the following publications:

1. Martincorena, I. *et al.* Somatic mutant clones colonize the human esophagus with age. *Science* **57,** eaau3879–14 (2018).

2. Martincorena, I. *et al.* Tumor evolution. High burden and pervasive positive selection of somatic mutations in normal human skin. *Science* **348,** 880–886 (2015).

Sample inclusion was determined by power calculations. We generated synthetic datasets of a given sizes, where the ground truth was known, and tested our ability to recover the known parameters as a function of data size. This allowed us to determine the minimum number of mutations required per dataset to fit our model. The results of this analysis are shown in figures 2b&c. Details of the computational model used are discussed in the Methods section.

**Replicates**

- You should report how often each experiment was performed
- You should include a definition of biological versus technical replication
- The data obtained should be provided and sufficient information should be provided to indicate the number of independent biological and/or technical replicates
- If you encountered any outliers, you should describe how these were handled
- Criteria for exclusion/inclusion of data should be clearly stated
- High-throughput sequence data should be uploaded before submission, with a private link for reviewers provided (these are available from both GEO and ArrayExpress)

Please outline where this information can be found within the submission (e.g., sections or figure legends), or explain why this information doesn’t apply to your submission:

No experiments were performed.

Data inclusion criteria is as stated in the previous box (power calculations from synthetic data).

**Statistical reporting**

- Statistical analysis methods should be described and justified
- Raw data should be presented in figures whenever informative to do so (typically when N per group is less than 10)
- For each experiment, you should identify the statistical tests used, exact values of N, definitions of center, methods of multiple test correction, and dispersion and precision measures (e.g., mean, median, SD, SEM, confidence intervals; and, for the major substantive results, a measure of effect size (e.g., Pearson's r, Cohen's d)
- Report exact p-values wherever possible alongside the summary statistics and 95% confidence intervals. These should be reported for all key questions and not only when the p-value is less than 0.05.

Please outline where this information can be found within the submission (e.g., sections or figure legends), or explain why this information doesn’t apply to your submission:

We used a Maximum Likelihood approach to fit our interval dN/dS models to the data, as this is the most appropriate methodology for a mathematically analytically tractable problem. The process is described in detail in the methods. For the fits and inferred parameters we report the maximum likelihood value and 95% CI. These are included in all figures where appropriate and when selection coefficients are reported in the main text we report the ML value and its 95% CI.

For dN/dS values we used the dndscv tool (<https://github.com/im3sanger/dndscv>) which reports a maximum likelihood dN/dS estimate along with 95% CI based on a Poisson regression model. We also used the SSB-dnds (<https://github.com/luisgls/SSB_selection>) to check our inferences were not affected by the choice of dN/dS method.

For directly fitting the clone size distribution we used a Bayesian method as this allowed us to directly quantify the uncertainty when we had limited data points. We used the Bayesian Regression Modelling with Stan (<https://cran.r-project.org/web/packages/brms/index.html>) R package to perform the inferences. All details of the priors used, the number of chains and number of iterations as well as checks on convergence are reported in the methods. For all figures where we report the results of the posterior parameters we use median point estimates and 80% and 95% credible intervals. These are described in the figure legends where appropriate.

(For large datasets, or papers with a very large number of statistical tests, you may upload a single table file with tests, Ns, etc., with reference to sections in the manuscript.)

**Group allocation**

- Indicate how samples were allocated into experimental groups (in the case of clinical studies, please specify allocation to treatment method); if randomization was used, please also state if restricted randomization was applied
- Indicate if masking was used during group allocation, data collection and/or data analysis

Please outline where this information can be found within the submission (e.g., sections or figure legends), or explain why this information doesn’t apply to your submission:

Not applicable, data are from previous studies and we do not test differences across groups.

**Additional data files (“source data”)**

- We encourage you to upload relevant additional data files, such as numerical data that are represented as a graph in a figure, or as a summary table
- Where provided, these should be in the most useful format, and they can be uploaded as “Source data” files linked to a main figure or table
- Include model definition files including the full list of parameters used
- Include code used for data analysis (e.g., R, MatLab)
- Avoid stating that data files are “available upon request”

Please indicate the figures or tables for which source data files have been provided:

Code necessary to completely reproduce all figures is provided as a snakemake workflow here: <https://github.com/marcjwilliams1/dnds-clonesize>

All the data necessary to produce the figures are also included in the repository.
